# The Obesogenic Environment: Epigenetic Modifications in Placental Melanocortin 4 Receptor Gene Connected to Gestational Diabetes and Smoking

**DOI:** 10.3389/fnut.2022.879526

**Published:** 2022-04-29

**Authors:** Marica Franzago, Annamaria Porreca, Mario D’Ardes, Marta Di Nicola, Luciano Di Tizio, Marco Liberati, Liborio Stuppia, Ester Vitacolonna

**Affiliations:** ^1^Department of Medicine and Aging, School of Medicine and Health Sciences, “G. d’Annunzio” University, Chieti, Italy; ^2^Center for Advanced Studies and Technology, “G. d’Annunzio” University, Chieti, Italy; ^3^Laboratory of Biostatistics, Department of Medical, Oral and Biotechnological Sciences, “G. d’Annunzio” University, Chieti, Italy; ^4^Department of Obstetrics and Gynaecology, SS. Annunziata Hospital, “G. d’Annunzio” University, Chieti, Italy; ^5^Department of Psychological, Health and Territorial Sciences, School of Medicine and Health Sciences, “G. d’Annunzio” University, Chieti, Italy

**Keywords:** obesity, nutritional health, gestational diabetes, DNA methylation, MC4R, maternal smoke exposure, fetal programming

## Abstract

**Background:**

Maternal metabolic insults as well as Gestational Diabetes Mellitus (GDM) influence the fetal health and may affect ‘offspring’s susceptibility to chronic diseases *via* epigenetic modifications. GDM, the most common metabolic disorder in pregnancy, can be considered the result of complex interactions between genetic and environmental factors. A critical point in this view is the identification of genes which are epigenetically modified under the influence of GDM. The melanocortin 4 receptor (*MC4R)* gene plays a crucial role in nutritional health by suppressing appetite and participating in energy control regulation. The correlations between pregnant ‘women’s metabolic profiles and placental epigenetic modifications of this gene have been poorly investigated.

**Objective:**

The aim of this study was to evaluate the effect of GDM and maternal clinical parameters at the third trimester of pregnancy to DNA methylation levels in the placenta at CpG sites of *MC4R* gene.

**Design and Methods:**

Socio-demographic and clinical characteristics, Mediterranean diet adherence, smoking habits, and physical activity were assessed at the third trimester of pregnancy of 60 Caucasian pregnant women, of which 33 with GDM. Clinical parameters of the newborns were recorded at birth. *MC4R* DNA methylation on maternal and fetal sides of the placenta was analyzed using bisulfite pyrosequencing.

**Results:**

*MC4R* DNA methylation levels at CpG1 and CpG2 were lower on the fetal side of the placenta in GDM-affected women than in non-GDM-affected recruits (*p* = 0.033). Moreover, DNA methylation levels on the maternal side at CpG1 were positively related to glucose concentration at 2-h oral glucose tolerance test (OGTT). On the other hand, CpG2 DNA methylation was positively related to both 1-h and 2-h during OGTT. Maternal DNA methylation level at CpG2 was also associated with low density lipoprotein cholesterol (LDL-C) at the third trimester of pregnancy (rho = 0.340, *p* < 0.05), while CpG1 methylation was negatively related to maternal weight variations at delivery (rho = −0.316, *p* < 0.05). Significant associations between *MC4R* DNA methylation on the maternal side and lipid profile at third trimester of pregnancy in women smokers were found.

**Conclusion:**

Our results suggest that *MC4R* methylation profile in the placenta is related to maternal metabolic and nutritional conditions, potentially affecting fetal programming and the future metabolic health of the newborn.

## Introduction

Gestational Diabetes Mellitus (GDM) is the most common condition of pregnancy with a global prevalence of 1 to 28% depending on the diagnostic criteria adopted (1), thus representing a major public health concern. GDM is the result of complex interactions between genetic and environmental factors. As GDM is strongly associated with both long- and short-term adverse maternal effects (i.e., pre-eclampsia, cesarean section, type 2 diabetes (T2D), and cardiovascular disease) as well as with offspring outcomes (i.e., macrosomia, birth trauma, neonatal hypoglycemia, metabolic syndrome, cardiovascular disease), the prevention, diagnosis and treatment of this condition are very much needed. As concerning offspring outcome, recent literature suggests that epigenetic alterations induced by maternal environmental and lifestyle, including hyperglicemia, unhealthy nutrition, tobacco smoking, alcohol consumption, environmental pollutants, endocrine disruptions, psychological stress, can influence fetal gene activity independently from the DNA sequence, highlighting the harmful consequences of different *in utero* exposures (2–5).

In this context, the epigenetics of GDM currently represents a very promising field of investigation in relation to the growing evidence of the role played by epigenetic alterations in the development of metabolic disturbances and their transgenerational effects (6). Therefore, epigenetic mechanisms, especially DNA methylation may play an important role not only in in fetal metabolic programming and chronic disease onset in adults, since hypermethylation of the CpG islands in the promoter region of a gene usually induces transcriptional silencing, while hypomethylation is related to transcriptional activation (5).

Since the placenta is metabolically very active and is responsive to environmental changes, it provides an optimal tissue to assess the molecular impact of GDM on childhood health. Previous literature data show that GDM leads to alteration in DNA methylation patterns of key energy metabolism-related genes, including adiponectin (*ADIPOQ*), leptin, (*LEPL*), ATP-binding cassette transporter A1 (*ABCA1*), and lipoprotein lipase (*LPL*) (7–12). Since these genes are mainly involved in energy balance as well as carbohydrate and lipid metabolism, placental epigenetic adaptations may have the potential to induce changes in the offspring’s metabolism and consequently shape their susceptibility to metabolic diseases later in life. A clear example of the concept of fetal metabolic programming through epigenetic changes is provided by the study of Gagné-Ouellet et al. (10), in which placental DNA methylation alterations were associated with GDM and body composition at 5 years of age.

On the other hand, to the best of our knowledge, no studies have investigated the melanocortin 4 receptor (*MC4R)* gene methylation profiles on the maternal and fetal sides of the placenta in GDM.

Among numerous genes associated with human obesity, the *MC4R* has proved to be particularly significant, by controlling appetite and playing a crucial role in the regulation of energy homeostasis, glucose metabolism, and body weight (13, 14). In addition, *MC4R* is linked to other aberrant feeding behaviors and obesity-related comorbidities, including binge eating disorder, cardiovascular disease, and hypertension (15–18). MC4R is a seven-transmembrane, G-protein–coupled receptor, primarily expressed in the central nervous system, particularly in the paraventricular nucleus and the arcuate nucleus of the appetite-regulating center in the hypothalamus (19). The activation of MC4R results in the inhibition of food intake, while loss of MC4R function in humans and rodents leads to obesity, insulin resistance, and diabetes (20, 21). A few specific mutations in the *MC4R* gene cause monogenic obesity, while other variants located within this gene or in its vicinity have been associated with eating behavior patterns, higher risk of obesity, cardiovascular risk factors and metabolic phenotypes (22–28).

Due to the regulatory network of *MC4R*, further insight into the epigenetic marks of this gene could be significant for a better understanding of metabolic disorders etiology. The study of the epigenetic modifications in placental tissues of GDM-affected patients may contribute to clarify the correlation between the exposure to an altered intrauterine environment and fetal metabolic programming. Taking the above into consideration, in the present study, we investigated the DNA methylation profiles in the placenta at CpG sites of the *MC4R* gene of women with and without GDM. In addition, we evaluated the correlation between maternal metabolic profiles at the third trimester of pregnancy and methylation of *MC4R*.

## Materials and Methods

### Study Design and Participants

Sixty caucasian pregnant women (age 33.9 ± 5.1 years) attending the Diabetes, Nutrition, and Metabolism Unit and the Obstetrics and Gynaecology Clinic, “G. d’Annunzio” University-Hospital “SS Annunziata” of Chieti, were recruited. Women with a positive history of drug or alcohol abuse, pre-gestational type 1 or 2 diabetes, overt diabetes, as well as other chronic diseases (such as malignancy, hypercholesterolemia) were excluded.

The GDM diagnosis was performed following the International Association of Diabetes and Pregnancy Study Groups (IADPSG) criteria (29). Thirty-three women had GDM, whereas twenty-seven were normoglycemic (NGT). The study was approved by the Ethics Committee of the “G. d’Annunzio” University, Chieti-Pescara. In compliance with the Declaration of Helsinki, all subjects provided written informed consent before their inclusion in the study.

During the first visit, data on demographic characteristics were collected. Anthropometric parameters were measured and recorded according to standard procedures. Clinical parameters including blood glucose, lipid profile [total cholesterol (TC), high-density lipoprotein cholesterol (HDL-C), low density lipoprotein cholesterol (LDL-C), triglycerides (TG)], and blood pressure were recorded at the third trimester. Specific attention was devoted to the record of smoke habits of the women. Physical activity was assessed using a short version of the International Physical Activity Questionnaire (IPAQ), and Adherence to the Mediterranean diet (Med-Diet) was evaluated through a validated 14-item questionnaire (PREDIMED) (30, 31). Clinical information about the newborns, including mode of delivery, gestational age, sex, and anthropometric measurements, was collected at birth.

### Placenta Tissue Sampling

Placenta biopsies of 0.5 cm^3^ were taken on the fetal and maternal sides within a few minutes after delivery and placenta expulsion in all women. In brief, placental biopsies were washed in PBS 1X and kept in RNAlater Stabilization Solution (Thermo Fischer Scientific, Waltham, MA, United States) at −80°C until nucleic acid extraction. DNA was purified using MagPurix 12s AutomatedNucleic Acid Purification System (Zinexts Life ScienceCorp., Taiwan) according to the manufacturer’s instructions, as previously reported (32).

### Epigenetic Analysis (DNA Methylation Analysis)

DNA methylation levels at CpG sites were assessed using pyrosequencing (Qiagen, Pyromark Q96; Diatech Italy). In brief, genomic DNA was treated with sodium bisulfite (NaBis) using the EpiTect Plus DNA Bisulfite Kit (33) (Qiagen), converting unmethylated cytosines to uracils. After bisulfite treatment, DNA (∼50 ng) was amplified by PCR using the Pyromark PCR kit (200) (Qiagen) and pyrosequenced according to manufacturer’s recommendations. The conditions in the PCR stage were 95°C for 15 min, 45 cycles at 94°C for 30 s, 60°C for 30 s, and 72°C for 30 s and a final extension at 72°C for 10 min. The PCR and pyrosequencing primers for the two CpGs tested within the *MC4R* gene (18q21.32) were MC4R F: 5′-AGGGTGATATAGATTTAGATGTAGAAGT-3′, MC4RR:5′[Btn]AAACAATATACTTTCCATTTCATTTTACAC -3′ (220 bp), and MC4RSeq: 5′- GTAGAAGTTTTTGAAGT TTG-3′ (2CpGs) as previously reported (34).

### Statistical Analysis

Data were summarized with absolute frequency (n) and column percentage (%) or mean and standard deviations (SD) when appropriate. Continuous variables were tested for normal distribution with Shapiro–Wilks’s test. Spearman rank correlation coefficient (rho)estimate the relationship between DNA methylation levels at CpG1 and CpG2 on the fetal and maternal side of the placenta and oral glucose tolerance test (OGTT) at baseline after 60 min and after 120 min. Pearson’s χ^2^ test was performed to compare for qualitative variables and unpaired *t*-test to assess mean differences.

To assess the potential confounding effect of insulin treatment, the non-parametric Mann-Whitney U test was applied. Furthermore, to identify possible confounding effects of the BMI on DNA methylation levels, we performed different multivariate linear regression models. We used the DNA methylation levels on maternal and fetal sides of the placenta as dependent variables, including two groups factor (GDM vs. NGT) as covariate and adjusting for BMI.

Moderation analysis is used to examine if the effect of an independent variable on the dependent variable is the same across different levels of another independent variable (moderator).

The simple slope analysis was employed to test moderating effects of smoking habit on the CpG1 and CpG2 methylation into the relationship with lipid profile and BMI in the pre-pregnancy period. All statistical tests were 2-sided with a significance level set at *p* < 0.050. All analyses were performed with the open-source statistical R environment (version 3.4.3, the R Foundation for Statistical Computing, Vienna, Austria).

## Results

The glucose tolerance status of the 60 recruits is summarized in Table 1. Thirty-three women had GDM (mean age 34.6 ± 4.1 years), whereas 27 were normoglycemic (NGT) (mean age 33.0 ± 6.1 years). Among GDM women, 27 were provided with a diet, while 6 were treated with both insulin and diet.

**TABLE 1:** Clinical characteristics of normoglycemic (NGT) and gestational diabetes (GDM) women expressed as mean and standard deviation (SD) and absolute frequency (n) and column percentage (%).

Characteristics	NGT (*n* = 27)	GDM (*n* = 33)	*p-value*
Age (years), mean (SD)	33.0 (6.1)	34.6 (4.1)	*0.242^+^*
Systolic blood pressure (mmHg), mean (SD)	108 (12.6)	114 (10.8)	*0.053^+^*
Diastolic blood pressure (mmHg), mean (SD)	65.8 (8.6)	73.2 (8.5)	***0.002*** *^+^*
PREDIMED, n (%)			*0.662* **^§^**
No adherence	1 (3.7)	2 (6.4)	
Medium adherence	17 (63.0)	22 (71.0)	
Maximum adherence	9 (33.3)	7 (22.6)	
IPAQ, n (%)			*0.332* **^§^**
Low	17 (63.0)	17 (56.7)	
Moderate	7 (25.9)	12 (40.0)	
High	3 (11.1)	1 (3.3)	
Smoking history, n (%)			*0.331* **^§^**
Non-smoker	24 (88.9)	25 (75.8)	
Smoker	3 (11.1)	8 (24.2)	
Pre-pregnancy weight (Kg), mean (SD)	60.7 (11.0)	72.9 (18.0)	***0.002*** *^+^*
Pre-pregnancy BMI (Kg/m^2^), mean (SD)	22.7 (2.9)	26.7 (6.7)	***0.003*** *^+^*
Weight at the end of pregnancy (Kg), mean (SD)	72.0 (12.2)	83.3 (19.6)	***0.009*** *^+^*
BMI at the end of pregnancy (Kg/m^2^), mean (SD)	26.9 (3.3)	30.6 (7.4)	***0.014*** *^+^*
Weight variation (Kg), mean (SD)	12.1 (4.4)	10.4 (5.4)	*0.190^+^*
Delivery, n (%)			***0.030^§^***
Vaginal delivery	22 (81.5)	14 (50.0)	
Cesarean section	5 (18.5)	14 (50.0)	
Third-trimester TC (mg/dl), mean (SD)	249 (50.8)	257 (53.1)	*0.666^+^*
Third-trimester HDL-C (mg/dl), mean (SD)	66.0 (20.0)	71.2 (16.6)	*0.455^+^*
Third-trimester TG (mg/dl), mean (SD)	217 (63.8)	203 (65.7)	*0.566^+^*
Third-trimester LDL-C (mg/dl), mean (SD)	148 (32.3)	145 (47.3)	*0.770^+^*
OGTT (mg/dl) at baseline (min), mean (SD)	79.0 (5.7)	89.2 (8.5)	<*0.001^+^*
OGTT (mg/dl) after 60 min, mean (SD)	119 (21.3)	168 (32.6)	<*0.001^+^*
OGTT (mg/dl) after 120 min, mean (SD)	96.7 (20.6)	137 (25.9)	<*0.001^+^*
First quarter fasting blood glucose (mg/dl), mean (SD)	77.8 (9.3)	85.0 (15.6)	*0.090^+^*

*Statistically significant values are in bold. ^§^ p-value derived from Chi squared test, + p-value derived from unpaired t-test.*

Patients with GDM showed higher BMI when compared to controls both in the pre-pregnancy period (26.7 ± 6.7 vs. 22.7 ± 2.94, *p* = 0.003) and at the end of pregnancy (30.6 ± 7.43 vs. 26.9 ± 3.35, *p* = 0.014). They also showed higher weight both in the pre-pregnancy (60.7 ± 11.0 vs. 72.9 ± 18.0, *p* = 0.002) and at the end of pregnancy (72.0 ± 12.2 vs. 83.3 ± 19.6, *p* = 0.009). Finally, they showed higher diastolic blood pressure (73.2 ± 8.55 vs. 65.8 ± 8.6, *p* = 0.002) and had a higher occurrence of cesarean section (*p* = 0.030) than controls. Regarding lifestyle habits, no differences, either in the physical activity or the MedDiet adherence between the groups, were found. Other comparisons between GDM and controls groups were not significant.

The main neonatal anthropometric characteristics are shown in Table 2. Mean gestational age at delivery was statistically significantly lower in the GDM group (*p* = 0.001). No other differences were found between the two groups.

**TABLE 2:** Neonatal outcomes relative to normoglycemic (NGT) and gestational diabetes (GDM) women expressed as mean and standard deviation (SD) absolute frequency (n) and column percentage (%).

Characteristics	NGT (*n* = 27)	GDM (*n* = 33)	*p-value*
Gestational week, mean (SD)	39.8 (1.0)	38.8 (1.1)	***0.001^+^***
Gender, n (%)			*0.917* **^§^**
Male	11 (40.7%)	15 (45.5%)	
Female	16 (59.3%)	18 (54.5%)	
Birth weight (grams), mean (SD)	3341 (418)	3324 (429)	*0.888* ***^+^***
Birth weight (percentiles), mean (SD)	51.0 (28.1)	63.6 (23.9)	*0.106* ***^+^***
One-minute Apgar scores, mean (SD)	8.58 (1.7)	8.85 (0.9)	*0.473* ***^+^***
Five-minute Apgar scores, mean (SD)	9.69 (1.0)	9.73 (0.7)	*0.875* ***^+^***
Birth head circumference (cm), mean (SD)	34.3 (1.7)	34.7 (1.0)	*0.323* ***^+^***
Birth length (cm), mean (SD)	51.0 (1.8)	50.0 (1.8)	*0.053* ***^+^***

*Statistically significant values are in bold. ^§^ p-value derived from Chi squared test, ^+^ p-value derived from unpaired t-test.*

### DNA Methylation Analysis of the Placental Melanocortin 4 Receptor Gene

Placental *MC4R* gene methylation was measured at 2 CpGs located at −200 and −212 from the transcription start site, respectively.

**FIGURE 1:**
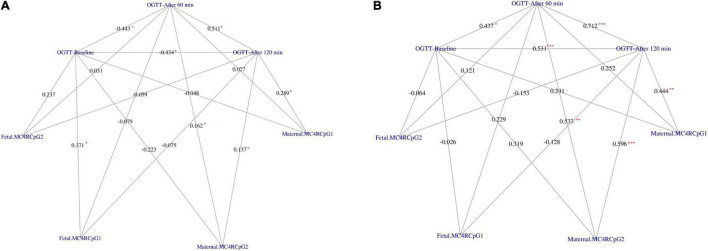
Correlation network between OGTT glucose concentration at baseline, after 60 min and after 120 min with the CpG1 and CpG2 at the maternal and fetal side of placenta in GDM **(A)** and Controls **(B)**, respectively. The weight on the edges indicates the Spearman rank correlation coefficient (rho), the red color star indicates statistically significant correlation coefficient. Significance code: “*” = *p* < 0.050, “**” = *p* < 0.010, “***” = *p* < 0.001.

Placental melanocortin 4 receptor DNA methylation levels were lower on the fetal side of GDM placentas as compared to controls (*p* = 0.033) (Table 3). Moreover, DNA methylation on the maternal side was correlated to 1-h and 2-h OGTT glucose concentrations at CpG2 in GDM and control groups (Figure 1). In addition, DNA methylation on the maternal side was also positively related to 2-h OGTT glucose concentrations at CpG1 in both groups (Figure 1). No difference between DNA methylation level on maternal and fetal side of placenta in GDM women treated with diet alone versus those treated with both diet and insulin was observed.

**TABLE 3:** *MC4R* DNA methylation levels on the maternal and fetal side of placenta normoglycemic (NGT) and gestational diabetes (GDM) women expressed as mean and standard deviation (SD). Mean differences were evaluated by unpaired *t*-test.

Characteristics	NGT (*n* = 27)	GDM (*n* = 33)	*p-value*
**Maternal side of placenta**			
CpG1	4.40 (3.9)	5.40 (4.8)	*0.379*
CpG2	14.6 (11.0)	17.6 (10.7)	*0.296*
Maternal Mean Methylation levels	9.50 (7.2)	11.5 (7.0)	*0.285*
**Fetal side of placenta**			
CpG1	6.4 (4.30)	4.7 (4.6)	*0.141*
CpG2	18.9 (10.0)	13.2 (9.5)	***0.028***
Fetal Mean Methylation levels	12.7 (6.7)	8.95 (6.3)	***0.033***

*Statistically significant values are in bold. ^+^p-value derived from unpaired t-test.*

Moreover, multivariate linear regression model shows that BMI could not be considered a confounder of relationship between groups and DNA methylation levels (data not shown).

Moreover, maternal DNA methylation level at the CpG2 was also associated with LDL-C at the third trimester of pregnancy (rho = 0.340, *p* < 0.050) while CpG1 methylation was negatively related to maternal weight variations at delivery (rho = −0.316, *p* < 0.050).

**FIGURE 2:**
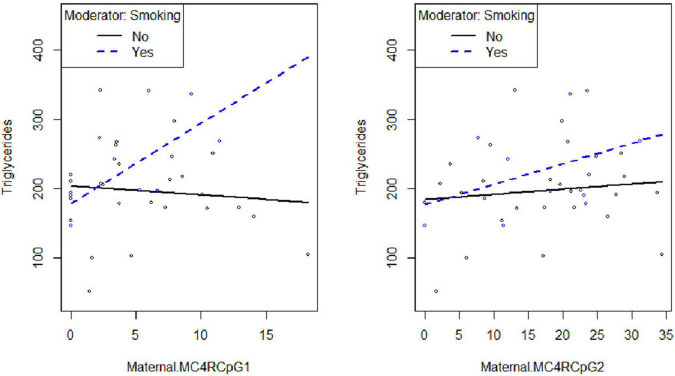
Smoking habit moderates the relation between *MC4R* DNA methylation at CpG1on the maternal side and TG at the third trimester of pregnancy (β_smoking=yes^* CpG1 DNA methylation_ = 12.94, *p* = 0.026).

**FIGURE 3:**
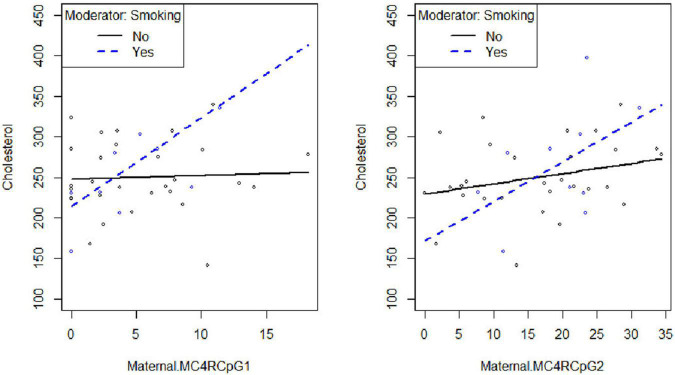
Smoking habit moderates the relation between *MC4R* DNA methylation at CpG1 on the maternal side and TC at the third trimester of pregnancy (β_smoking=yes^* CpG1 DNA methylation_ = 10.49, *p* = 0.036).

**FIGURE 4:**
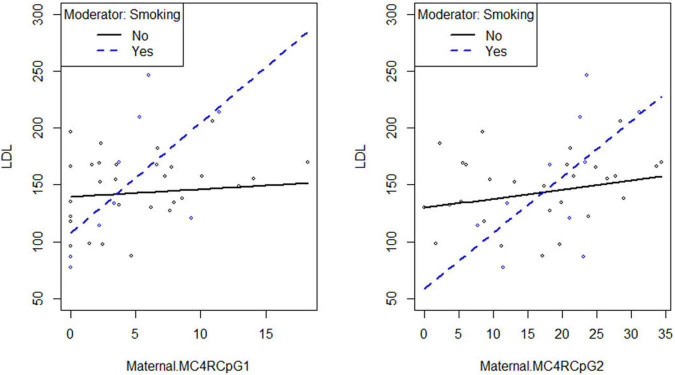
Smoking habit moderates the relation between *MC4R* DNA methylation at CpG1 on the maternal side and LDL at the third trimester of pregnancy (β_smoking=yes^* CpG1 DNA methylation_ = 9.08, *p* = 0.027).

Smoking habit influences the relation between CpG1 DNA methylation on the maternal side and TG levels at the third trimester of pregnancy (β_smoking=yes^* CpG1 DNA methylation_ = 12.94, *p* = 0.026) (Figure 2), as well as between CpG1 DNA methylation and TC (β_smoking=yes^* CpG1 DNA methylation_ = 10.49, *p* = 0.036) (Figure 3). In addition, it was observed a statistically significant interaction effect for LDL (β_smoking=yes^* CpG1 DNA methylation_ = 9.08, *p* = 0.027) (Figure 4) but not for HDL (β_smoking=yes^* CpG1 DNA methylation_ = −1.35, *p* = 0.450). These interactions were observed both in smoking and not smoking women.

## Discussion

To the best of our knowledge, the present study investigates, for the first time, DNA methylation profiles of the *MC4R* gene on both the maternal and the fetal side of the placenta in pregnant women with and without GDM. Our results showed that the methylation profile at the CpG1 and CpG2 sites was significantly lower on the fetal side of placenta in GDM women when compared with controls.

We found that GDM women had a significantly greater BMI in both periods, pre-pregnancy and at the end of pregnancy, which was consistent with previous studies. Even considering BMI as a potential confounder, the significance of the results persisted.

A higher *MC4R* DNA methylation levels on the maternal side were associated with both higher maternal LDL-C levels at the third trimester of pregnancy and higher maternal glucose concentrations 1 and 2 h OGTT. In addition, *MC4R* methylation levels on the maternal side was inversely related to maternal weight variations at delivery in all women.

Increasing evidence has indicated that exposure to adverse maternal factors in pregnancy may trigger short- and long-term complications for the newborn, potentially impairing metabolic health in adult life *via* epigenetic mechanisms (6). In this view, great attention has been devoted to identification of genes involved in this process. *MC4R* was selected since the associations between variants in this gene with obesity, T2D, and eating behavior have been widely studied (17, 18), and recently it has been reported to be differentially methylated in spermatozoa of lean subjects as compared to obese patients (35), as well as in vegans as compared to omnivores (34).

It has been shown that defects in MC4R can lead to a clinical phenotype defined by lack of satiety and early-onset obesity (36–39). Moreover, Morgan et al. (14) demonstrated that MC4R signaling differentially regulates a set of different physiological functions and glucose homeostasis regardless of energy balance, as evidenced also by data on animal models where the deletion of *Mc4r* gene in both sympathetic and parasympathetic cholinergic neurons impaired glucose homeostasis, leading to hyperinsulinemia and modest insulin resistance (40, 41). In this context, previous observations have shown that MC4R-agonism enhances peripheral insulin sensitivity and improves glucose tolerance in rodents and non-human primates (42, 43). Taken together, all these observations raise the question about possible *MC4R* placental epigenetic alterations induced by GDM during pregnancy and the potential increased risk of alterations in glucose homeostasis in the offspring. As a matter of fact, results obtained in the present study evidenced a decreased in *MC4R* DNA methylation levels on the fetal side of the placenta in GDM women. Regarding our results about the correlation between *MC4R* DNA methylation levels and maternal LDL-C levels, it is newsworthy to note that besides having a prominent role in metabolic regulation, it has been suggested that MC4R signaling pathways may affect sympathetic activity and cardiovascular physiology (44). Interestingly, MC4R-null mice showed elevated TG levels and abnormal expression of genes related to fatty acid (FA) synthesis, suggesting an important role of the MC4R in regulating liver FA metabolism (45). In this context, Kwon et al. (46, 47) showed an association between the *MC4R* methylation pattern in the cord blood and the metabolic profiles in childhood, especially with regard to the TG level, suggesting that DNA methylation alterations occurring early in life may affect metabolic profiles in childhood (48).

Another interesting point is the one related to maternal smoke. Prenatal smoking exposure has some effects on energy metabolism (33) and causes several adverse health outcomes, including the development of metabolic disorders in adulthood (49). Although evidence from human and animal studies suggested that cigarette smoking during pregnancy influences DNA methylation patterns in mother and offspring (50), the potential pathways mediating nicotine’s effects *via* epigenetic mechanisms are still unclear.

We previously demonstrated that differences in the methylation patterns of *FTO* gene occur in the placental DNA of women exposed to tobacco smoke during pregnancy (32). As a consequence, in the present study, we aimed to analyze the consequences of maternal smoking on placental DNA methylation of the *MC4R* gene as well. Our results show that *MC4R* methylation on the maternal side of placenta was significantly increased in women who smoked during pregnancy, who also showed elevated lipid profile at the third trimester. These results apparently appear to disagree with previous literature data showing that smoking suppresses appetite *via* the Mc4r activation in rodents (51), as well as with more recent study of Yi et al., demonstrating that the expression of Mc4r is not affected by *in utero* cigarette smoke exposure (52). However, both these studies were carried out on rat neurons, not on placenta. On the other hand, present findings are consistent with our previous studies, which emphasized the correlation between lipid parameters and smoke during pregnancy along with the identification of genetic and epigenetic markers (32, 53, 54).

It should be noted that one of the limitations of the present study is that gene expression analysis has not been performed in placenta biopsies.

In summary, our study suggests that a common maternal exposure such as smoking may induces placental methylation modifications at *MC4R* gene, in turn inducing alterations in lipid parameters. It should be noted that the mechanisms underlying the complexity of multiple exposure-outcome relationships are poorly understood, partly due to the dynamic nature of these relationships across the lifecycle (55, 56). As a result, the effects of pre-conceptional and perinatal exposures to insults need to be clarified. It is necessary to improve our understanding of which early-life metabolic exposures may be the most critical for reprogramming the growth trajectories of offspring (55). Furthermore, the molecular mechanisms occurring in the intrauterine period, which is the most sensitive time for the establishment of epigenetic variability, remain largely unexplored.

The most relevant question is whether or not the evidenced relationship between placental DNA methylation at *MC4R* and maternal conditions may interfere with the metabolic health programming of the newborn. Actually, results obtained on the fetal side of the placenta samples make this question more difficult to answer. In fact, the fetal side of the placenta showed a reduced *MC4R* methylation, rather than an increased one such as in the maternal side. This could suggest that some sort of adaptation mechanism could take place in order to preserve the health of the offspring. This can be confirmed by the evidence that neonatal parameters in the analyzed sample do not show differences between the offspring of GDM and controls mothers. However, it is known that downstream melanocortin-responsive circuits responsible for different physiological actions do diverge, making it crucial an ongoing effort to better understand melanocortin pathways and their myriad roles in metabolic disease (57). Thus, the possible impact of the epigenetic alterations on lipid and glucose metabolism impairments later in life needs to be further evaluated, and further studies are needed to evaluate the effects of maternal insults as well as epigenetic alterations during critical periods of development (such as pregnancy) and their consequences in adult life. In this view, *MC4R* DNA methylation levels will need to be assessed in these children over time, in order to determine whether the association between DNA methylation at this locus observed in the placenta and the carbohydrate and lipid metabolic clinical parameters does persist throughout infancy and childhood.

## Data Availability Statement

The original contributions presented in the study are included in the article/supplementary material, further inquiries can be directed to the corresponding author.

## Ethics Statement

The studies involving human participants were reviewed and approved by Ethics Committee of the “G. d’Annunzio” University, Chieti-Pescara. The patients/participants provided their written informed consent to participate in this study.

## Author Contributions

EV and MF designed the study and contributed to the interpretation of results. EV and LD contributed to the recruitment of patients and to clinical evaluation. MF conducted the experiments. EV, ML, and MD’A contributed to data acquisition. AP performed the statistical analysis. MDN advised on the statistical approach. MF, EV, and LS drafted the manuscript. EV, MDN, and LS are the guarantors of this work. All authors contributed to the article and approved the submitted version.

## Conflict of Interest

The authors declare that the research was conducted in the absence of any commercial or financial relationships that could be construed as a potential conflict of interest.

## Publisher’s Note

All claims expressed in this article are solely those of the authors and do not necessarily represent those of their affiliated organizations, or those of the publisher, the editors and the reviewers. Any product that may be evaluated in this article, or claim that may be made by its manufacturer, is not guaranteed or endorsed by the publisher.
